# Assessing medical education: the challenge of measuring complex competencies

**DOI:** 10.1016/j.clinsp.2026.101205

**Published:** 2026-10-22

**Authors:** Newton Kara-Junior, Eduardo Motta

**Affiliations:** Faculdade de Medicina da Universidade de São Paulo, São Paulo, SP, Brazil

The publication of the results of the Enamed (National Examination for the Assessment of Medical Training) in 2025 revealed that approximately 13,000 newly graduated physicians that year in Brazil, performed unsatisfactorily on the assessment of learning. This finding shed light on a longstanding concern: the insufficient quality of medical education provided by a substantial proportion of Brazilian medical schools. Beyond quantifying an educational deficit, these results brought the need for more effective mechanisms to assess medical training to the national agenda and led the Brazilian federal government to consider the possibility of a mandatory licensing examination for medical practice.^1^

Once the need to assess medical graduates has been acknowledged, an equally important question arises: how should such assessment be conducted? From a technical perspective, there are strong arguments that a test consisting of only 100 multiple-choice questions, as initially proposed, has important limitations in assessing the breadth of competencies established by Ministry of Education Ordinance No. 478, which introduced the Common Reference Framework for the Assessment of Medical Training.^2^

The first concern relates to insufficient content sampling. The framework encompasses seven major areas of medicine, 21 content categories, 15 general competencies, different healthcare settings within the Brazilian Unified Health System (Sistema Único de Saúde, SUS), and three learning domains — cognitive, practical, and attitudinal. In this context, a 100-item examination would provide, on average, approximately 14 questions per major area, and even fewer items for each specific set of competencies. This represents a limited sample of the extensive and complex construct being assessed. Importantly, however, the absolute number of test items is not necessarily the key issue; what matters is the number of observations available for each domain and context. Well-constructed multiple-choice questions, particularly those based on clinical vignettes, can provide meaningful assessments of several components of clinical reasoning, judgment, applied ethics, and decision-making.

The second concern involves construct validity. The framework does not restrict itself to theoretical knowledge. It encompasses competencies such as communication, procedural skills, teamwork, professionalism, self-reflection, medical documentation, and shared decision-making. Multiple-choice questions can assess, with reasonable precision, pattern recognition, clinical reasoning, interpretation of diagnostic tests, and selection of appropriate management strategies. However, this format cannot directly demonstrate whether a student is able to perform a procedure appropriately, communicate difficult news, conduct a patient-centered consultation, work effectively within a team, or demonstrate professional behavior in real-life situations. In terms of Miller’s pyramid, a written examination primarily assesses what a candidate “knows” and, to some extent, “knows how,” but it cannot directly demonstrate “shows how” or “does”.^3^

A third issue concerns the relationship between reliability and breadth of assessment. The greater the number and diversity of competencies one seeks to infer, the greater the number of independent observations of performance that are required. A 100-item examination may provide reasonable precision in estimating general cognitive knowledge, but it is unlikely to support equally reliable inferences about dozens of competencies distributed across multiple clinical areas and practice settings. The inevitable consequence is a reduction in the depth and representativeness of the assessment.

Another frequently underestimated issue is the washback effect of assessment on the curriculum. In educational systems, what is assessed in high-stakes examinations tends to influence what schools and students prioritize and teach. If a national examination consists exclusively of multiple-choice questions, there will be a natural incentive to focus preparation on test-taking and question-solving strategies. The risk is that this approach will divert time and effort from practical training, clinical communication, professionalism, and supervised learning in real-world settings. Paradoxically, an instrument designed to assess medical education could contribute to narrowing the curriculum precisely when the Ordinance proposes a broader, competency-based approach.^4^^,^^5^

An interesting alternative is programmatic assessment, which does not attempt to infer competence from a single examination but instead builds an assessment based on multiple observations of performance over time, using different instruments, assessors, and clinical settings. Objective tests can assess knowledge and aspects of clinical reasoning; structured examinations can assess practical skills and communication; and workplace-based assessments can evaluate performance with real patients. The final judgment of competence is then based on the integration of multiple sources of evidence rather than on the isolated result of a single examination.^6^

Thus, there is no single instrument capable of measuring the full spectrum of medical competence. Rather, there is a system of assessment capable of gathering sufficient evidence to support an informed judgment about competence. The fundamental question should therefore be: *What set of evidence do we need before we can conclude that an individual is prepared to practice medicine?* A national examination may have substantial value as a mechanism for accountability, benchmarking, and identification of systemic deficiencies, but it should not be mistaken for a comprehensive certification of professional competence.

This discussion leads to an even deeper issue: the crisis in Brazilian medical education is not fundamentally a problem caused by the absence of a national examination, but by deficiencies in the educational processes that precede graduation.

The rapid expansion of medical education in Brazil has occurred without proportional growth in the availability and quality of teaching hospitals, clinical training sites, qualified preceptors, clinical supervision, and robust longitudinal assessment mechanisms.^7^ In this context, a national 100-item multiple-choice examination may be useful for assessing one component of the cognitive knowledge of graduating physicians. However, it is unlikely, by itself, to provide a solution to the structural problems affecting the quality of medical education.

In other words, an examination may identify some of the consequences of the mass expansion of medical education, but it is unlikely to address its underlying causes. High-quality medical education depends on diverse and supervised clinical experiences, continuous competency-based assessment, direct observation of performance, systematic feedback, and the progressive development of professional identity. A written examination can and should complement this process, but it cannot replace it.

There is, therefore, an apparent contradiction that must be addressed: Portaria No. 478 proposes a model of medical education based on broad and complex competencies, while the current discussion focuses on an assessment instrument potentially limited to measuring knowledge through objective questions. If the goal is to determine whether future physicians are genuinely prepared to care for patients, assessment must address not only what they know, but also what they can do and how they do it.

In this regard, longitudinal and programmatic assessment throughout medical school, combining theoretical and practical instruments, direct observation of performance, assessment across different healthcare settings, and longitudinal monitoring of competency development, appears to be more consistent with the proposed competency-based model of medical education.

The central question, therefore, should not simply be, *“What will the examination look like?”* but rather, “What do we need to ensure when we state that a physician is prepared to practice medicine?” The answer to this question should guide the design of the assessment system — and, above all, the model of medical education that Brazil intends to provide to society.

## Conflicts of interest

The authors declare no conflicts of interest.

## Data Availability

All data are available from the corresponding author upon reasonable request.
